# Quantitative ^18^F-fluorocholine positron emission tomography for prostate cancer: correlation between kinetic parameters and Gleason scoring

**DOI:** 10.1186/s13550-017-0269-0

**Published:** 2017-03-21

**Authors:** Joshua D. Schaefferkoetter, Ziting Wang, Mary C. Stephenson, Sharmili Roy, Maurizio Conti, Lars Eriksson, David W. Townsend, Thomas Thamboo, Edmund Chiong

**Affiliations:** 1 A*STAR-NUS Clinical Imaging Research Centre, Centre for Translational Medicine (MD6), 14 Medical Drive, #B1-01, Singapore, 117599 Singapore; 2 Department of Diagnostic Radiology, National University Hospital, Singapore, Singapore; 3 Department of Urology, National University Health System, Singapore, Singapore; 4 Siemens Healthcare Molecular Imaging, Knoxville, TN 37919 USA; 5 Department of Pathology, National University Health System, Singapore, Singapore

**Keywords:** PET, Choline, Histology, Prostate cancer, Tracer kinetic modeling

## Abstract

**Background:**

The use of radiolabeled choline as a positron emission tomography (PET) agent for imaging primary tumors in the prostate has been evaluated extensively over the past two decades. There are, however, conflicting reports of its sensitivity and the relationship between choline PET imaging and disease staging is not fully understood. Moreover, relatively few studies have investigated the correlation between tracer uptake and histological tumor grade. This work quantified ^18^F-fluorocholine in tumor and healthy prostate tissue using pharmacokinetic modeling and stratified uptake parameters by histology grade. Additionally, the effect of scan time on the estimation of the kinetic exchange rate constants was evaluated, and the tracer influx parameters from full compartmental analysis were compared to uptake values quantified by Patlak and standardized uptake value (SUV) analyses.

^18^F-fluorocholine was administered as a 222 MBq bolus injection to ten patients with biopsy-confirmed prostate tumors, and dynamic PET data were acquired for 60 min. Image-derived arterial input functions were scaled by discrete blood samples, and a 2-tissue, 4-parameter model accounting for blood volume (2T4k+Vb) was used to perform fully quantitative compartmental modeling on tumor, healthy prostate, and muscle tissue. Subsequently, all patients underwent radical prostatectomy, and histological analyses were performed on the prostate specimens; kinetic parameters for tumors were stratified by Gleason score. Correlations were investigated between compartmental *K*_1_ and *K*_i_ parameters and SUV and Patlak slope; the effect of scan time on parameter bias was also evaluated.

**Results:**

Choline activity curves in seven tumors, eight healthy prostate regions, and nine muscle regions were analyzed. Net tracer influx was generally higher in tumor relative to healthy prostate, with the values in the highest grade tumors markedly higher than those in lower grade tumors. Influx terms from Patlak and full compartmental modeling showed good correlation within individual tissue groups. Kinetic parameters calculated from the entire 60-min scan data were accurately reproduced from the first 30 min of acquired data (*R* ^2^ ≈ 0.9).

**Conclusions:**

Strong correlations were observed between *K*_i_ and Patlak slope in tumor tissue, and *K*_1_ and SUV were also correlated but to a lesser degree. Reliable estimates of all kinetic parameters can be achieved from the first 30 min of dynamic ^18^F-choline data. Although SUV, *K*_1_, *K*_i_, and Patlak slope were found to be poor differentiators of low-grade tumor compared to healthy prostate tissue, they are strong indicators of aggressive disease.

**Electronic supplementary material:**

The online version of this article (doi:10.1186/s13550-017-0269-0) contains supplementary material, which is available to authorized users.

## Background

Prostate cancer (PCa) is common in the USA and is the second leading cause of cancer death in men. In 2015, 220,800 American men were diagnosed and 27,540 are expected to die from the disease [1]. Despite its prevalence, prognoses are generally good for this disease but accurate diagnoses are critical, as the therapeutic options depend on the aggressiveness and potential progression of the disease.

Clinically, most prostate cancers are detected first by a prostate-specific antigen (PSA) blood test or digital rectal exam and are confirmed through subsequent biopsy. Once confirmed, the patient will undergo diagnostic imaging procedures to assess the spread of the disease and plan treatment. Common imaging modalities include transrectal ultrasound, MRI, and PET/CT. MRI sessions generally consist of T1- and T2-weighted, DWI (diffusion-weighted imaging), and sometimes DCE (dynamic contrast enhanced) and spectroscopy sequences.

Molecular imaging with PET has several benefits due to the ability to target specific underlying biological processes like increased metabolism, upregulated protein and phospholipid synthesis, expression of androgen receptors and membrane proteins, or the osteoblastic reaction adjacent to metastases in the bone marrow [2]. Clinically, routine PET imaging is performed with ^18^F-fluorodeoxyglucose, but this has shown limited utility for detecting PCa tumors [3, 4]. Improvements in tumor uptake have been reported for other tracers including ^11^C- and ^18^F-choline [3, 5–7], ^11^C-acetate [8–10], ^11^C-methionine [11, 12], and ^18^F-fluorodihydrotestosterone [13, 14]. However, these tracers have demonstrated relatively low specificity due to uptake in normal prostate tissue and benign lesions including prostatitis, high-grade intraepithelial neoplasia, or benign prostatic hyperplasia (BPH) [15, 16]. Consequently, recent focus has shifted to monitoring the expression of prostate-specific membrane antigen (PSMA), in which elevated levels have been histologically correlated with PCa tumor progression [17], androgen independence [18], and metastasis [19]. PET agents have been developed as radiolabeled PSMA analogs like ^68^Ga-PSMA [20, 21] or the PSMA inhibitors ^18^F-DCFBC [22–24] and ^18^F-DCFPyL [25]. These new, specific tracers, with high tumor to background contrast, are extremely promising and are being used more frequently, particularly for distant metastases and recurrence. Notwithstanding the growing interest in these novel tracers, ^18^F-Fluorodexoxyglucose (FDG) and choline are still the most clinically used PET tracers clinically for prostate cancer.

Choline has a strong uptake in the primary prostate tumor, even though it has not been shown to be highly specific, since benign prostatic hyperplasia also shows high uptake. Nevertheless, PET imaging of high choline uptake could be used to guide or support biopsy and surgery of primary prostate cancer. Biopsy procedures, especially of prostate tissue, can cause many issues including patient discomfort and infection. False negative results are also common, which may lead to a repeat biopsy and incorrect diagnosis. Incorporating imaging at an earlier point in the clinical routine can potentially improve patient management.

Radiolabeled choline has been used as a PET agent in humans for 20 years [26], and the prostate was among the first imaging targets [27]. Over the years, many studies have been conducted investigating various aspects of its clinical utility including its efficacy for detecting primary tumors and systemic involvement [28, 29], preoperative staging and pelvic lymphadenopathy [30–32], post-therapy biochemical recurrence and correlations with PSA [33, 34], performance relative to FDG and other PET tracers [3, 35], MRI [36, 37] and MR spectroscopy [38], and quantitative evaluations of tracer kinetics in tissue [39–41]. Choline standardized uptake value (SUV) has been studied extensively, and there are many studies reporting the performance of choline for detecting patients with biopsy-confirmed prostate tumors, though with somewhat controversial results concerning the reported detection sensitivities [16, 42]. However, there are only a limited number of studies which have correlated choline uptake with histological tumor grade, in terms of histologic grading and Gleason score [28, 43, 44]. There are even fewer studies which have modeled the tracer kinetics and investigated those relationships with tissue grade [40] and none to our knowledge using the ^18^F-labeled derivative.

The aim of this study is to investigate the pharmacokinetics of ^18^F-fluoromethylcholine in various tissues, including pathology-confirmed primary prostate tumors and healthy prostate tissue, and perform a lesion-based correlation of quantified choline uptake with tumor grade. In particular, compartmental modeling was performed, and tissue parameters were stratified by Gleason score. Additionally, the relationships between tracer perfusion/extraction and influx compartmental rate constants were evaluated between SUV and Patlak linear regression analyses. The effect of scan time on kinetic parameter bias was also assessed.

While the immediate purpose of this work is to investigate the correlation of choline uptake kinetics with histologic tumor grade, it also provides the validation of methods that could be used with other tracers such as PSMA, which may offer an improved approach for characterizing and staging primary prostate cancer.

## Methods

Ten men with biopsy-confirmed prostate cancer were recruited for the study. The mean age was 67 years (range, 59–72 years). Blood samples were not collected for one patient (patient 9), and so, he was not included in the kinetic analyses.

The imaging session included a PET/MR scan on the Biograph mMR (Siemens Medical Solutions Inc.). After the imaging session (18.7 ± 12.6 days), each patient underwent a radical prostatectomy and the excised prostate was sliced at 4–5 mm intervals before undergoing tissue processing. At each interval, thin sections of the paraffinized tissue slices were obtained by microtomy and stained for light microscopic assessment.

### Image acquisition

For PET/MR, subjects were positioned on the scanning bed, and a 60-min dynamic PET acquisition of the pelvis was started simultaneously with the intravenous injection of 222 MBq ^18^F-choline. The radiotracer was administered as a 1 mL bolus infused at a rate of 3 mL/s followed by a 20 mL saline flush. Venous blood samples were taken periodically over the course of the PET scan. The nurse’s visits into the scanning room to draw the blood were timed to accommodate the various MR sequences, so as to avoid potential field disturbances during the MR acquisitions. Efforts were made to sample the blood at least every 10 min.

The listmode PET data were divided into 30 frames of 12 × 10, 6 × 30, 5 × 60, 4 × 300, and 3 × 600 s duration, and each frame was reconstructed using an ordinary Poisson ordered subset expectation maximization (OP-OSEM) algorithm, with point spread function (PSF), with 3 iterations and 21 subsets. Attenuation correction was performed on the emission data using a multi-point T1-weighted DIXON sequence to derive the PET 511 keV attenuation map. The final image volume matrices of 172 × 172 × 127, with voxel size of 4.173 × 4.173 × 2.031, were post-smoothed with a 5-mm Gaussian filter.

### Arterial input function

**Fig. 1:**
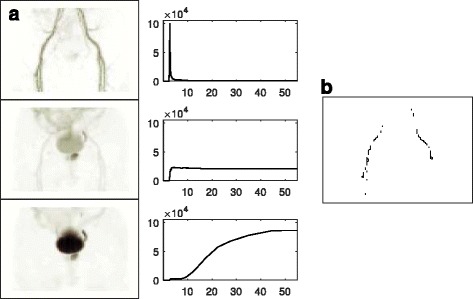
Maximum intensity projection factor images representing arterial, prostate, and bladder components, with corresponding time-activity curve signals (**a**). The voxels with the highest values in the vascular factor image corresponded to the iliac and femoral structures and defined the VOI for extracting the arterial input curve (**b**). The first and last ten slices were omitted from the factor analyses to avoid edge noise effects

The arterial blood signal was extracted from the iliac and femoral vessels in the image frames using factor analysis [45, 46], which involved the calculation of the covariance matrix for all dynamic patient voxels. Eigenanalysis was performed on the covariance matrix and the eigenvectors were ranked according to their corresponding eigenvalues. The eigenvector corresponding to the blood signal was always included as one of the strongest, i.e., having one of the largest eigenvalues, because the initial bolus peak yielded a temporal profile which was uniquely different from the other tissue curves. Factor image volumes were then generated by associating to each voxel, a value representing its contribution to each signal. Figure 1 shows the maximum intensity projections of factor image volumes for one patient. Typically, the strongest three signals comprised the arterial blood, prostate, and bladder activity curves.

The top 40% of the voxels showing the strongest contribution to the arterial signal defined the 3D volume of interest (VOI) over the iliac and femoral arterial structures, and this was projected back on the original image frames to extract the corresponding activity curve. The image-derived blood curve was corrected for partial volume and spillover to produce the actual arterial whole-blood activity *A*, which was calculated with the following formula:

$$ A = \left({A}_0-{a}_1* T\right)/{a}_2 $$

Here, *A*_0_ is the original, uncorrected arterial blood curve, as measured in the images, and *T* is the unknown background tissue activity, approximated by a nearby region in muscle. The coefficients *a*_1_ and *a*_2_ correspond to spillover and partial volume, respectively. A least-squares fitting algorithm was employed to find the best estimates of these coefficients, while fitting the extracted blood curve data to the manual blood samples.

**Fig. 2:**
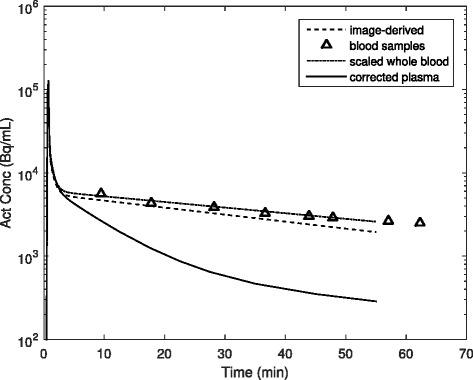
Example of arterial ^18^F-choline activity concentration curves. The image-derived blood activity (*dashed line*) was taken from the iliac and femoral vessels. The scaled whole-blood curve (*dash-dot line*) was found by correcting the image-derived curve for partial volume and tissue spillover while simultaneously scaling it to the manual venous blood samples (*triangles*). The plasma concentration (*solid line*) was calculated by applying the plasma partitioning coefficient and metabolite correction to the scaled whole-blood activity

Blood-plasma partitioning and metabolite corrections were applied to the whole-blood activity curve to calculate the plasma concentration for the active tracer. As intracellular choline uptake occurs in blood cells, the relative plasma to whole-blood activity ratio changes with time. This general trend was modeled by applying the same plasma-to-whole blood ratio relationship to all patients, starting with 1.3 at injection and decreasing linearly to 1.1 at 60 min (Adriaan Lammertsma, personal communication, July 20, 2016). The plasma fractions were measured in the first four patients, and the approximation was found to be reasonable. In this work, the plasma parent fraction was not measured; the metabolite curve applied to all patients was similar to that found in previous work [47]. An example of the input curve fitting is shown in Fig. 2 for a patient with eight manual blood samples. Additionally, the blood sampling data for the first four patients are included in Additional file 1: Figure S1.

### PET analyses and kinetic modeling

Regions of normal and focal tracer uptake were identified in the prostate and normal uptake in muscle tissue, and 30% threshold VOIs were drawn in the last frame of the dynamic series, i.e., images over 50–60 min. This threshold was found to provide acceptable delineation for all tumor foci, and if bladder expansion caused significant movement of the prostate, the VOIs were reregistered in each frame. The tissue time-activity data were weighted by frame length and fit using a 2-tissue model to estimate four compartmental rate parameters and blood volume fraction (*V*_B_). For the kinetic modeling and batch analyses, a combination of PMOD Kinetic Modeling Tool version 3.6 (PMOD Technologies Ltd.) and in-house developed software was used.

Selected prostate regions were confirmed histologically as tumor and healthy tissue, and the corresponding perfusion-related parameter *K*_1_ and tracer influx parameter for each, defined as

$$ {K}_{\mathrm{i}}=\frac{K_1*{k}_3}{k_2+{k}_3}, $$

were correlated with respective 60-min SUV and Patlak analysis collectively for all tissue groups. Additionally, *K*_i_ was correlated with SUV and Patlak within each tissue group separately.

### Histologic correlation

**Fig. 3:**
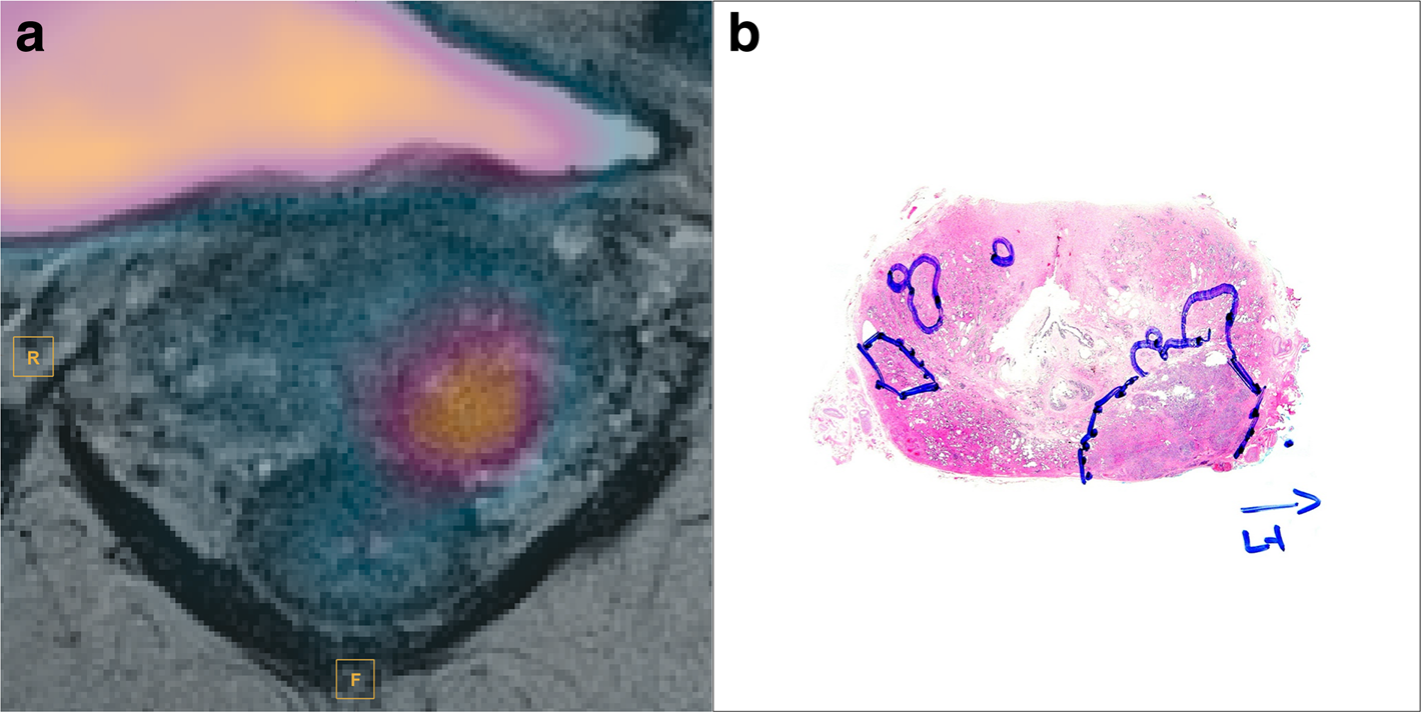
^18^F-Choline PET frame at 12.5 min fused with T2-weighted image (**a**). Focal tracer uptake is seen in the left lateral lobe. Histology confirmed tumor at this region with Gleason score 4 + 3 (**b**). No visible uptake was seen in the contralateral, lower grade tumor tissue (Gleason score 3 + 4)

The excised prostate glands were fixed in 4% buffered formalin before undergoing gross dissection. The formalin-fixed prostate specimens were cut into slices of approximately 4 mm thickness, with each slice undergoing tissue processing in its own cassette. After tissue processing, the prostatic tissue was embedded in paraffin in large blocks (megablocks) and sectioned at 3 μm thickness. The sections were mounted on large slides and were stained with hematoxylin and eosin (H&E) before undergoing microscopic evaluation. The tumor tissue was delineated on each slide and assigned modified Gleason grades and an overall Gleason score [48]—this is illustrated in Fig. 3 for a tumor with Gleason score 4 + 3.

The diagnosis of prostatic adenocarcinoma in each case was made by application of well-known microscopic criteria [49]. Prostatic adenocarcinoma can have a variety of microscopic patterns, and these patterns also contribute to the grade of the tumor. In general, prostatic adenocarcinoma tends to consist of malignant acini which are smaller than the adjacent benign acini, and which tend to infiltrate between these benign acini. The malignant acini may also have irregular or elongated contours and may fuse into cribriform structures or solid sheets of cells, among other patterns.

In addition to the routine clinical work-up, the pathologist was asked to follow a sector-based reporting scheme, consistent with the radiologist PI-RADS report. Tumor regions, with their associated Gleason score, were manually delineated within the anatomical map, facilitating the direct coregistration between regions of interest in PET and histopathology; this approach was similar to that used in previous works [40, 43, 44].

## Results

Focal ^18^F-choline uptake was identified in eight prostates, which were confirmed tumors by histology with 3 having Gleason score 3 + 4 (though one of these was from patient 9, without blood sampling, and was omitted from the analyses), 1 with 3 + 5, and 4 with 4 + 3. These and regions of healthy prostate tissue in eight patients were delineated with threshold VOIs. Tumors were not visualized in two patients by PET—histology reports showed the volumes of the largest tumors were less than 0.05 cm^3^ in both cases. Hence, the uptake trends of the tumors in these patients were not analyzed. For comparison to non-prostatic tissue, muscle activity curves were also measured for all patients.

**Fig. 4:**
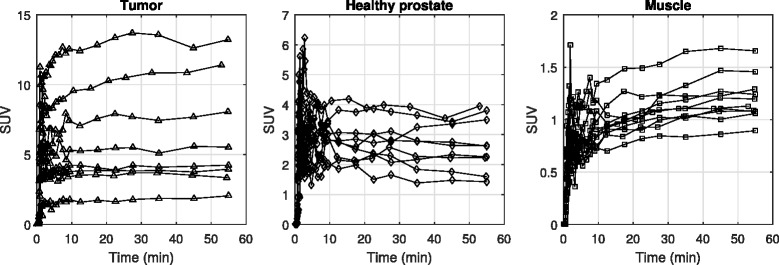
Tissue time-activity curves shown for all included tissue regions, eight in tumor, nine in healthy prostate, and ten in muscle. Tissue curves for tumors plateaued or increased throughout the duration of the scan, but in most cases, those for healthy prostate peaked within the first 5 min, then plateaued or decreased. Muscle activity curves were much lower but always showed slight increases with time. Curves were time-shifted to match plasma input peaks and each plot shows a different ordinate scale in order to highlight tissue trends. For this and subsequent plots, tumors are respresented by triangles, healthy prostate by diamonds and muscle by squares

Overall patterns in tissue uptake were identified. ^18^F-choline activity curves were relatively high in prostatic tumors, and all curves in tumor tissue either plateaued around 5 min or showed slightly increasing uptake throughout the scan. Uptake also peaked early in healthy prostate tissue, but the curves generally plateaued early or showed slight decrease throughout the scan. This trend was observed in all nine prostates, except for one, which showed only slightly increasing activity. For all measured prostate regions, ^18^F-choline was rapidly transported from the vascular to intracellular space and metabolized—the majority of the tissue uptake occurred within the first 3 min. Extraction to muscle was also quick, but, in all cases, the tissue activity curves increased continuously throughout the scan. Muscle uptake was much lower than tumor and healthy prostate. All time-activity curves included in the analyses are shown in Fig. 4.

Fully quantitative PET analyses using a 2-tissue compartmental model, accounting for blood volume, were performed on all tissue activity curves over 60 min. The individual values of *K*_1_–*k*_4_ for all tissue regions are given in Table 1. The standard errors on the parameter estimation, derived from the covariance of the model Jacobian at the solution, are also given in the table—they are expressed as the percentage of the corresponding parameter values. The standard error defines the confidence in the estimation precision; a large error may imply that the imposed model is not appropriate for the data. However, even when the errors were high on the individual parameters, the corresponding errors on the net influx macroparameters were generally low because of interparameter correlation. Mean compartmental influx parameters were 0.28 ± 0.21 for the tumors, 0.11 ± 0.04 for the healthy prostates, and 0.04 ± 0.01 for muscle.

**Table 1:** 2T4k+Vb model rate parameter values (with %SE) in muscle, healthy prostate, and tumor tissue

	Muscle				Healthy prostate				Tumor			
Patient	*K*_1_	*k*_2_	*k*_3_	*k*_4_	*K*_1_	*k*_2_	*k*_3_	*k*_4_	*K*_1_	*k*_2_	*k*_3_	*k*_4_
1	0.04 (21.8%)	0.05^a^ (>100%)	1.14^a^ (>100%)	0^a^ (>100%)	0.16 (39.2%)	0.39^a^ (>100%)	0.66 (80%)	0.01 (66.5%)	0.25 (5.5%)	0.17 (23.3%)	0.23 (13.7%)	0.01 (9.8%)
2	0.05 (10.7%)	0.08^a^ (>100%)	0.83^a^ (>100%)	0^a^ (>100%)	0.32 (6%)	0.18 (15.5%)	0.11 (10.7%)	0.01 (14.7%)	–	–	–	–
3	0.05 (2.4%)	0.18 (11.2%)	0.33 (5.5%)	0 (31.8%)	0.17 (4%)	0.05 (13.5%)	0.02 (50.8%)	0.01^a^ (>100%)	0.44 (13.8%)	0.11 (62.1%)	0.16 (40.1%)	0.01 (30.6%)
4	0.05 (4.5%)	0.16 (21%)	0.3 (10.3%)	0^a^ (>100%)	0.22 (14.5%)	0.16 (37.7%)	0.09 (30.5%)	0.02 (27.3%)	0.34 (9.8%)	0.16 (27.1%)	0.12 (17.3%)	0 (38.2%)
5	0.05 (23.9%)	0^a^ (>100%)	2.7^a^ (>100%)	0.4^a^ (>100%)	0.18 (28%)	0.04^a^ (>100%)	0.23^a^ (>100%)	0^a^ (>100%)	–	–	–	–
6	0.04 (61.8%)	0^a^ (>100%)	4.9^a^ (>100%)	1.73^a^ (>100%)	0.37 (7.4%)	0.15 (22.4%)	0.13 (15.7%)	0.01 (13.7%)	0.62 (5%)	0.11 (31.2%)	0.26 (17.2%)	0.01 (15.6%)
7	0.07 (4.8%)	0.11 (38%)	0.4 (19%)	0^a^ (>100%)	0.27 (15.4%)	0.17 (52%)	0.18 (30.1%)	0.01 (25.6%)	0.29 (2.7%)	0.19 (8.4%)	0.18 (4.6%)	0.01 (6%)
8	0.07 (5%)	0.13 (39.7%)	0.42 (22.3%)	0.02 (16.3%)	–	–	–	–	1.14 (18.52%)	0.13 (71.2%)	0.2 (44.8%)	0.01 (27.9%)
10	0.1 (15.2%)	0.25 (35.9%)	0.16 (18.6%)	0^a^ (>100%)	0.13 (17.1%)	0.11 (72.6%)	0.16 (45.7%)	0.01 (38.5%)	0.38 (6.7%)	0.2 (16.1%)	0.11 (10.2%)	0.01 (21.9%)

^a^Individual parameter estimates were not stable in the cases where the 2T4k+Vb model was not optimal

Patlak analyses were also performed on the same time-activity curves and SUVs recorded. Patlak linear regression fits used the dynamic data after 10 min, and SUV values were calculated by multiplying the tissue activity in the last (50–60-min) frame by the patient weight and dividing by the time-corrected injection activity. Patlak slope and SUV were 0.22 ± 0.15 and 6.6 ± 3.2 for the tumors, 0.09 ± 0.04 and 2.8 ± 0.8 for the healthy prostates, and 0.04 ± 0.01 and 1.2 ± 0.16 for muscle, respectively. The numbers for each patient are given in Table 2.

**Table 2:** Various metrics (with %SE) from PET analyses of healthy prostate and tumor tissue

	Healthy prostate			Tumor			
Patient	Tracer flux	Patlak slope	60-min SUV	Tracer flux	Patlak slope	60-min SUV	Gleason score
1	0.1 (2.7%)	0.08 (3.2%)	2.9	0.15 (1.4%)	0.12 (3.1%)	4.3	3 + 4
2	0.12 (2.6%)	0.1 (3.2%)	2.8	–	–	–	–
3	0.04 (32.6%)	0.03 (12.1%)	1.4	0.26 (7.3%)	0.22 (2.5%)	7	4 + 3
4	0.08 (8.6%)	0.05 (5.4%)	3	0.14 (4.3%)	0.12 (2.6%)	6.2	4 + 3
5	0.15 (6.5%)	0.16 (3.9%)	3.8	–	–	–	–
6	0.17 (3.7%)	0.12 (5.6%)	3.7	0.44 (2.6%)	0.4 (1.5%)	10.7	4 + 3
7	0.14 (4.5%)	0.11 (3.7%)	2.8	0.14 (0.7%)	0.12 (1.6%)	2.9	3 + 5
8	–	–	–	0.7 (16.5%)	0.48 (3.4%)	10.9	4 + 3
10	0.08 (4.9%)	0.06 (3.4%)	2.3	0.14 (2.8%)	0.12 (1.7%)	4.2	3 + 4

**Fig. 5:**
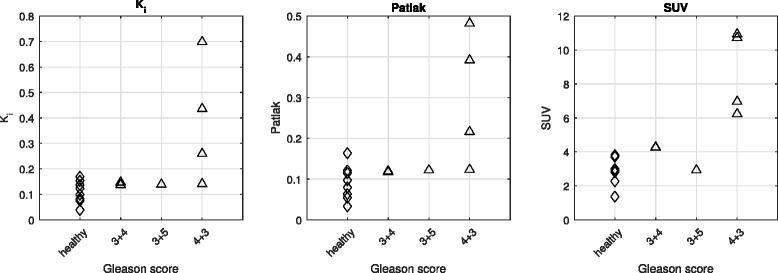
Choline uptake metrics in tumor and healthy prostate. Intrapatient choline uptake was significantly higher in tumors with Gleason score of 4 + 3

The tumor data in this table is represented graphically in Fig. 5, where the three key numerical parameters, *K*_1_, Patlak slope, and SUV, are plotted as a function of Gleason score.

**Fig. 6:**
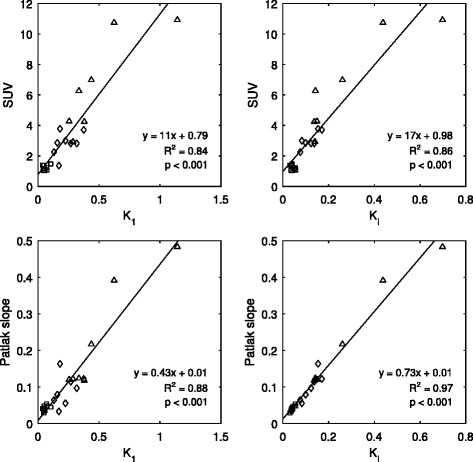
Correlation between fully quantitative compartmental modeling using *K*_1_ (*left plots*) or macroinflux parameter *K*_i_ (*right plots*) and simplified methods SUV (*row A*) and Patlak (*row B*) across all tissues. *K*_i_ showed better correlation with the simplified methods than *K*_1_. However, the good correlations observed between *K*_i_ and both SUV and Patlak were mainly attributed to the relatively large intertissue range of values used for the regression fit. Points corresponding to tumor activity are represented by *triangles*, those from healthy prostate tissue are *diamonds*, and muscle are *squares*

The correlations between SUV and Patlak analysis and compartmental tracer kinetic modeling were evaluated, using *K*_1_ and macroinflux parameter *K*_i_ as figures of merit. Correlation of the simplified methods with *K*_i_ was better than that with *K*_1_, shown in Fig. 6. The regression fit is displayed on each respective plot, along with the coefficient of determination and *p* value. Significance was calculated using a one-tailed *F* test against the null hypothesis of no correlation.

**Fig. 7:**
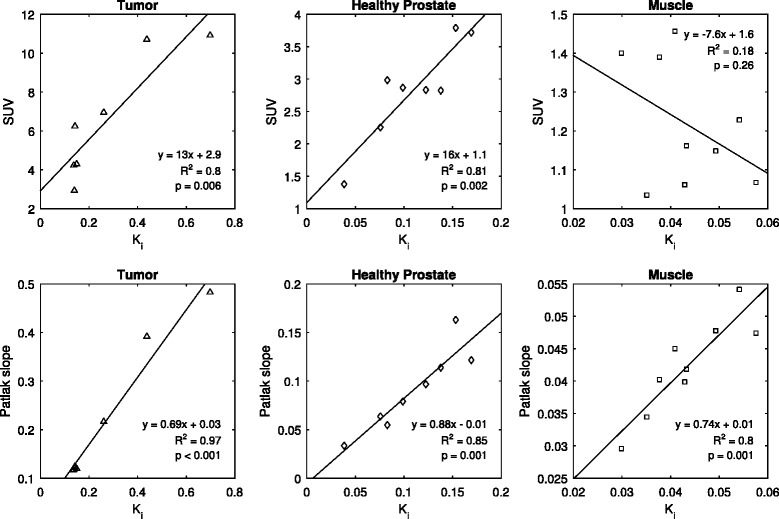
Correlation between influx parameter *K*_i_ and SUV (*row A*) was relatively poor within individual tissue groups compared to that in the total sampled tissue population, shown in Fig. 6. Good correlations were however, generally observed between compartmental modeling and Patlak slope (*row B*), showing the validity of a simplified Patlak model as a substitute for the more complex compartmental analysis

The good correlation observed was largely attributed to the relatively large intertissue range of values used for the regression fit, especially for SUV. Hence, the correlations were generally poorer within individual tissue groups. Patlak influx, however, still performed relatively well with respect to kinetic influx parameter *K*_i_, as seen in Fig. 7.

**Fig. 8:**
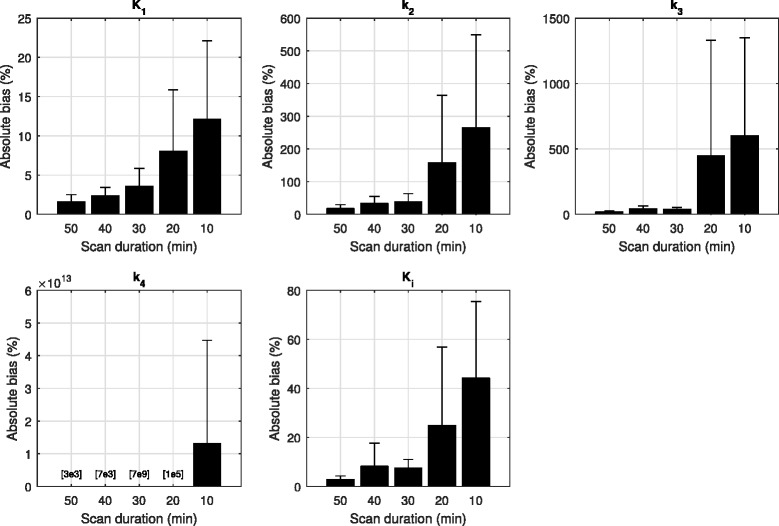
Absolute estimation bias on kinetic rate parameters of all tissue regions for various dynamic scan times, relative to those at 60 min. Shorter scan times introduced bias in estimates of parameters *k*_2_–*k*_4_. However, *K*_1_ and the macroinflux parameter *K*_i_ were relatively stable. For all parameters, 30 min of scan data seemed acceptable for accurate estimation

The effect of scan time on the estimation of the compartmental rate parameters was investigated. The absolute bias on the estimates of *K*_1_–*k*_4_ and *K*_i_ for all tissue regions, relative to those at 60 min, is shown in Fig. 8.

**Fig. 9:**
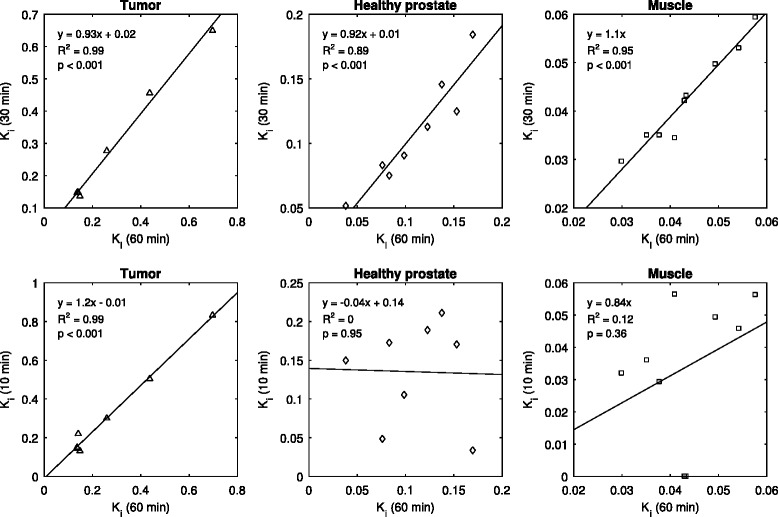
The choline influx parameter *K*_i_ values calculated for the entire 60-min scan were correlated with those calculated using only the first portion of dynamic data. Good correlation was still seen in all tissue groups for the 30-min data, with slopes near 1. The 10-min data showed relatively good correlation for tumor but poor correlation for healthy prostate and muscle

Relative bias on the *k*_2_–*k*_4_ parameter estimates was observed for the shorter scan times, but *K*_1_ and *K*_i_ showed more stability, with mean biases at 3.6 and 7.4% and 12.1 and 44.2% at 30- and 10-min scan times, respectively. The relationship between the *K*_i_ flux values from the 60-min scan and those calculated using only the first 30 and 10 min of dynamic data was investigated, and the correlation for each tissue group is shown in Fig. 9.

For all tissue groups, the data showed that choline influx parameters, calculated with 30 min of dynamic data, were significantly well correlated with those of the full 60 min.

## Discussion

This work investigated ^18^F-choline uptake patterns in prostatic tumors, healthy prostate tissue, and muscle; tracer kinetic analyses were performed with compartmental modeling. Plasma input functions were obtained through image-derived methods, fit to manual blood measurements while accounting for partial volume and spillover effects, and corrected for plasma partitioning and metabolites. Correlations were evaluated between the influx rate parameter calculated from fully quantitative analyses and Patlak and 60-min SUV.

A limitation of this study is that arterial blood was not collected from the subjects, so accurate measurements of the radiolabeled metabolites were not possible. Hence, we applied a single metabolite correction to all plasma curves. This method, however, is not ideal, since high interpatient variability in the parent tracer fraction has been reported [41] which could lead to errors and bias in the estimations of the rate parameters. To characterize these effects, we systematically ran the curve fitting algorithm on all tissue data with different metabolite corrections [41, 47, 50] and corresponding plasma inputs. We found that there were indeed rather large variances on some of the estimations of the individual microparameters *K*_1_–*k*_4_, but that the calculations of the macroflux parameter *K*_i_ were robust. A similar investigation was performed to assess the effect of the plasma partitioning. For the first four patients with manual blood sampling, the kinetic parameters estimated using the linear partitioning method used here were compared to those estimated using the mean of the individual patient measurements as a constant partitioning ratio. Again, we found that the estimates of the individual compartmental rate parameters were far more sensitive to this than the calculated tracer influx. Plots showing the results of both of these tests are shown in Additional file 1: Figures S2 and S3.

**Fig. 10:**
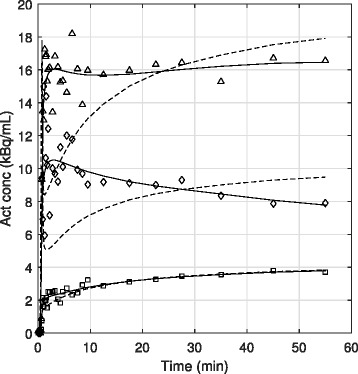
Comparison of different compartmental models, 1T1k+V_B_ is *dashed line* and 2T4k+V_B_ is *solid*. The 1-tissue model could not accurately fit the ^18^F-choline data in all tissue groups. Tumor data are *triangles*, healthy prostate data are *diamonds*, and muscle are *squares*

Regarding the choice of compartment model, we ran the curve fitting algorithm on all tissue data using four different models: 1T1k+Vb, 1T2k+Vb, 2T3k+Vb, and 2T4k+Vb. The most appropriate compartmental model to characterize choline kinetics was chosen based on the mean value of the Akaike information criterion (AIC) [51] calculated for all tissue curves (Additional file 1: Figure S4), and this was found to be the 2T4k+V_B_ compartmental model. However, the Akaike weight was only just the lowest with this model—in fact, half of the tissue curves preferred the 2T3k+Vb model, and different choices of input model may have led to small differences in this result. The use of a reversible model is not consistent with previous works which have reported irreversible tracer kinetics in tissue—indeed, the *k*_4_ values were found to be very small compared to the other parameters. So, even though the AIC analyses suggested a model which did not constrain *k*_4_ to be zero, the net influx rates were still well correlated with those of Patlak analyses. The 2T4k+V_B_ model performed well overall, but in some cases, it rendered the fitting algorithm unable to find the global minimum, especially in the muscle regions where the slow monotonically increasing uptake was better represented by an irreversible model. Fitting the data to a model with too many parameters produced unstable estimates on parameter values, as seen in Table 1. This is consistent with previous work which reported that the 2T3k+Vb model provided the lowest AIC, but a 1T1k+Vb compartmental model may be best to quantify ^18^F-fluoromethylcholine due to its robustness [41]. We were however unable to achieve reliable fits for all tissue data with this model, as illustrated in Fig. 10. This is probably due to the different regions investigated, since we found that different tissues may require different models, even within the same subject.

The parameters that were evaluated to characterize choline uptake in tissue were SUV, *K*_1_, *K*_i_, and Patlak slope. The most relevant result of this study is that SUV, *K*_1_, *K*_i_, and Patlak slope are indeed strong indicators of aggressive tumor in the prostate. As can be seen in Fig. 5, while all three parameters show limited correlation with Gleason score for low-grade tumors, they all show a dramatic increase at Gleason score 4 + 3. This could suggest that SUV, and even better *K*_i_ and Patlak slope, could be used as indicators of aggressive tumors. Microparameters *k*_2_, *k*_3_, and *k*_4_ showed no correlation with Gleason score (results not reported). However, it is possible that these kinetic parameters could be more helpful in PET imaging with tracers other than choline.

It is well known that choline is rapidly transported and phosphorylated within the cells [39], and indeed, we observed that the majority of the tissue tracer uptake occurred within the first 5–10 min. Hence, this work investigated the effects of scan time reduction on the quantitative analyses. Here, we found that the calculation variability in the microparameters *k*_2_–*k*_4_ was sensitive to the scan time but estimates of perfusion and choline transport, represented by *K*_1_, and the macroflux parameter *K*_i_ were more stable; this finding is not uncommon for compartmental modeling tasks. This notwithstanding, accurate characterizations of ^18^F-choline influx for all tissue could be quantified using 30 min of dynamic scan data, as can be appreciated in Figs. 8 and 9. Further, the *K*_i_ correlations we found were comparable to those reported by Verwer et al. for scan durations 40 vs. 10 min, 40 vs. 20 min, and 40 vs. 30 min. Our correlation slopes for the same times were 1.2, 1.1, and 0.99 and those from Verwer et al. were 0.93, 1.05, and 1.03. Our *R* ^2^ values were 0.93, 0.95, and 1, and those from Verwer et al. were 0.97, 0.9, and 1. These findings may prove useful for future studies investigating dynamic ^18^F-choline PET for prostate applications, since scan time should be kept to a minimum for patient convenience and scanner throughput considerations and also to avoid bladder filling issues (which is not a consideration for the ^11^C-labeled derivative). Motion is not typically a major problem with pelvic scanning, but expansion of the bladder can cause deformation of the internal structures, affecting the voxel measurements over time. Shorter scan times would minimize these effects.

An important result of this work is the development of a method and a protocol to perform kinetic studies of prostate cancer tracer in primary tumor and perform correlation between compartmental model parameters and Gleason score. We plan to apply these methods with other newer and more specific tracers for prostate cancer, with the purpose of correlating aggressiveness of the disease and PET imaging parameters, therefore providing a non-invasive method for supporting staging and therapy planning of primary tumor.

## Conclusions

This study of the kinetics of ^18^F-choline, using both Patlak method and fully compartmental modeling, showed a strong correlation between *K*_i_ and Patlak slope in tumor tissue; *K*_1_ and SUV were also correlated to a lesser degree. Due to differences in ^18^F-choline uptake profiles for different tissues, a 2-tissue model was needed to quantify tracer uptake. Fully quantitative analyses were performed for accurate characterization of the tissue uptake, but this work showed that reliable estimates of all kinetic parameters can be achieved with only 30 min of dynamic data. Finally, an interesting result of this study is that even if SUV, *K*_1_, *K*_i_, and Patlak slope are poor differentiators of low-grade tumor compared to healthy prostate tissue, they are strong indicators of aggressive disease: they all dramatically increase at Gleason score 4 + 3. The good correlation between *K*_i_ as determined from the compartment model and from the Patlak analyses indicates that the Patlak plot can be used as a substitute for full compartmental analysis of the ^18^F-choline data in the prostate.

## Additional file

Additional file 1: Figure S1. Venous blood sampling points for four patients. These data were consistent with the plasma partitioning model applied to all subjects in this work. **Figure S2.** Compartmental model rate parameters were independently estimated using five different metabolite correction models (a). Mean values are shown for all tissue regions in all patients (b); diamonds represent regions of healthy prostate and triangles represent tumors—with different colors corresponding to different Gleason scores. Error bars show the standard deviation of the results obtained with the four metabolite corrections. Calculations of the macroinflux parameters were more robust to changes in the input profile than were the individual compartmental parameters. **Figure S3.** Compartmental model rate parameters were independently estimated using two different plasma partitioning models for the first four patients with manual blood sampling; diamonds represent regions of healthy prostate and triangles represent tumors—with different colors corresponding to different Gleason scores. Error bars show the standard deviation of the results obtained with the two partitioning methods. Similar to the results shown in Additional file 1: Figure S2, estimations of choline influx rates were less sensitive than were the individual parameters. **Figure S4.** Akaike information criterion analysis for all tissue regions. Four different compartmental models were used to fit the data and the best model was determined by the lowest total AIC score. This plot shows mean AIC values in each of the three tissue regions; error bars show the interpatient standard deviation in each tissue category. The 2T4k+vB model (slightly) yielded the lowest overall AIC, but the reversible *k*_4_ parameters were generally small compared to the other parameter values. Good correlation was still observed between choline influx terms, calculated from this model and Patlak analyses. **Figure S5.** Example patient maximum intensity projection image shown with 3D delineated tissue regions: red is tumor, green is healthy prostate, and blue is muscle. (DOCX 260 kb)

## Abbreviations

2T4k+Vb: Compartmental model using 2-tissues and 4 rate + blood volume parameters
AIC: Akaike information criterion
BPH: Benign prostatic hyperplasia
DCE: Dynamic contrast enhanced
DSRB: Domain specific review board
DWI: Diffusion-weighted imaging
FDG: ^18^F-Fluorodeoxyglucose
H&E: Hematoxylin and eosin
OP-OSEM: Ordinary Poisson ordered subset expectation maximization
PCa: Prostate cancer
PET: Positron emission tomography
PSA: Prostate-specific antigen
PSF: Point spread function
PSMA: Prostate-specific membrane antigen
SUV: Standardized uptake value
V_B_: Blood volume fraction
VOI: Volume of interest
