# Erratum to: Quantitative 18F-fluorocholine positron emission tomography for prostate cancer: correlation between kinetic parameters and Gleason scoring

**DOI:** 10.1186/s13550-017-0299-7

**Published:** 2017-06-21

**Authors:** Joshua D. Schaefferkoetter, Ziting Wang, Mary C. Stephenson, Sharmili Roy, Maurizio Conti, Lars Eriksson, David W. Townsend, Thomas Thamboo, Edmund Chiong

**Affiliations:** 1 A*STAR-NUS Clinical Imaging Research Centre, Centre for Translational, Medicine (MD6), 14 Medical Drive, #B1-01, Singapore, 117599 Singapore; 2 Department of Diagnostic Radiology, National University Hospital, Singapore, Singapore; 3 Department of Urology, National University Health, System, Singapore, Singapore; 4 Siemens Healthcare Molecular Imaging, Knoxville, TN 37919 USA; 5 Department of Pathology, National University, Health System, Singapore, Singapore

## Erratum

Upon publication of the original article [1], it was noticed that the author “Joshua D. Schaefferkoetter” was incorrectly included as “Joshua J.D. Schaefferkoetter”.

## References

[1] Schaefferkoetter JD (2017). Quantitative ^18^F-fluorocholine positron emission tomography for prostate cancer: correlation between kinetic parameters and Gleason scoring. EJNMMI Research.

