# Supplementary material for: Quantitative 18F-fluorocholine positron emission tomography for prostate cancer: correlation between kinetic parameters and Gleason scoring
Source: EJNMMI Res. 2017 Mar 21;7:25. doi: 10.1186/s13550-017-0269-0 (PMC5360745; doi:10.1186/s13550-017-0269-0)
Supplement: Additional file 1: Figure S1. — Venous blood sampling points for four patients. These data were consistent with the plasma partitioning model applied to all subjects in this work. Figure S2. Compartmental model rate parameters were independently estimated using five different metabolite correction models (a). Mean values are shown for all tissue regions in all patients (b); diamonds represent regions of healthy prostate and triangles represent tumors—with different colors corresponding to different Gleason scores. Error bars show the standard deviation of the results obtained with the four metabolite corrections. Calculations of the macroinflux parameters were more robust to changes in the input profile than were the individual compartmental parameters. Figure S3. Compartmental model rate parameters were independently estimated using two different plasma partitioning models for the first four patients with manual blood sampling; diamonds represent regions of healthy prostate and triangles represent tumors—with different colors corresponding to different Gleason scores. Error bars show the standard deviation of the results obtained with the two partitioning methods. Similar to the results shown in Additional file 1: Figure S2, estimations of choline influx rates were less sensitive than were the individual parameters. Figure S4. Akaike information criterion analysis for all tissue regions. Four different compartmental models were used to fit the data and the best model was determined by the lowest total AIC score. This plot shows mean AIC values in each of the three tissue regions; error bars show the interpatient standard deviation in each tissue category. The 2T4k+vB model (slightly) yielded the lowest overall AIC, but the reversible k 4 parameters were generally small compared to the other parameter values. Good correlation was still observed between choline influx terms, calculated from this model and Patlak analyses. Figure S5. Example patient maximum intensity projection image shown with 3D d [file 13550_2017_269_MOESM1_ESM.docx]

**Figure S1.** Venous blood sampling points for 4 patients. These data were consistent with the plasma partitioning model applied to all subjects in this work.

B

A

**Figure S2.** Compartmental model rate parameters were independently estimated using 5 different metabolite correction models (A). Mean values are shown for all tissue regions in all patients (B); diamonds represent regions of healthy prostate and triangles represent tumors – with different colors corresponding to different Gleason scores. Error bars show the standard deviation of the results obtained with the 4 metabolite corrections. Calculations of the macro influx parameters were more robust to changes in the input profile than were the individual compartmental parameters.

**Figure S3.** Compartmental model rate parameters were independently estimated using 2 different plasma partitioning models for the first 4 patients with manual blood sampling; diamonds represent regions of healthy prostate and triangles represent tumors – with different colors corresponding to different Gleason scores. Error bars show the standard deviation of the results obtained with the 2 partitioning methods. Similar to the results shown in Supp. Figure 2, estimations of choline influx rates were less sensitive than were the individual parameters.

**Figure S4.** Akaike information criterion analysis for all tissue regions. Four different compartmental models were used to fit the data and the best model was determined by the lowest total AIC score. This plot shows mean AIC values in each of the 3 tissue regions; error bars show the interpatient standard deviation in each tissue category. The 2T4k+vB model (slightly) yielded the lowest overall AIC, but the reversible k_4_ parameters were generally small compared to the other parameter values. Good correlation was still observed between choline influx terms, calculated from this model and Patlak analyses.

**Figure S5.** Example patient maximum intensity projection image shown with 3D delineated tissue regions: red is tumor, green is healthy prostate, and blue is muscle.
